# Super-enhancers in esophageal carcinoma: Transcriptional addictions and therapeutic strategies

**DOI:** 10.3389/fonc.2022.1036648

**Published:** 2022-10-27

**Authors:** Yang Shi, Meiqi Wang, Dan Liu, Saif Ullah, Xing Ma, Huiyu Yang, Bingrong Liu

**Affiliations:** ^1^ Department of Gastroenterology, The First Affiliated Hospital of Zhengzhou University, Zhengzhou, China; ^2^ State Key Laboratory of Esophageal Cancer Prevention and Treatment, Zhengzhou University, Zhengzhou, China; ^3^ Academy of Medical Sciences Zhengzhou University, Zhengzhou, China; ^4^ Department of Nuclear Medicine, The Affiliated Cancer Hospital of Zhengzhou University & Henan Cancer Hospital, Zhengzhou, China

**Keywords:** super-enhancers, esophageal carcinoma, core regulatory circuitry, transcriptional dependence, histone acetylation

## Abstract

The tumorigenesis of esophageal carcinoma arises from transcriptional dysregulation would become exceptionally dependent on specific regulators of gene expression, which could be preferentially attributed to the larger non-coding *cis*-regulatory elements, *i.e.* super-enhancers (SEs). SEs, large genomic regulatory entity in close genomic proximity, are underpinned by control cancer cell identity. As a consequence, the transcriptional addictions driven by SEs could offer an Achilles’ heel for molecular treatments on patients of esophageal carcinoma and other types of cancer as well. In this review, we summarize the recent findings about the oncogenic SEs upon which esophageal cancer cells depend, and discuss why SEs could be seen as the hallmark of cancer, how transcriptional dependencies driven by SEs, and what opportunities could be supplied based on this cancer-specific SEs.

## Introduction

Esophageal carcinoma is the fourth most common gastrointestinal cancer worldwide. The incidence and mortality rates of esophageal carcinoma accounts for 11.75% and 14.99%, respectively (1). According to statistics from the International Researches Agency of Cancer (IRAC), the mortality rate of the cancer would increase 69% in China by 2040, while the main histological type is squamous cell carcinoma (ESCC) (2, 3). The standard-of-care regimens consisted of surgery, platinum-based chemotherapy, radiotherapy, and PD-1/PD-L1 blockade therapy (4, 5), which have improved the survival rate and patient’s quality of life. However, chemoradiotherapy resistance, postoperative recurrence and unresectable advanced lesions are still impeding the long-term survival of these patients. Given that the limited actionable drivers in ESCC, more effective avenues based on the clarification of the carcinogenesis are required. Recently, it is widely recognized that the disease-associated variations are bound up with enhancer regions, especially super-enhancers (SEs), which undoubtedly provides novel insights into therapeutic maneuvers.

Enhancers refer to non-coding part of genome that activate genes expression independent of orientation, distance, and location regarding its transcription start sites (TSSs). These regulatory elements always provide binding sites for multiple transcription factors (TFs) that can also be transcribed to produce non-coding enhancer RNAs (eRNAs). More recently, cluster of enhancers located genomic proximity were identified as the unique transcriptional single entity, known as SEs. SEs are underpinned by highly abundant and orchestrated interactions with transcription apparatus and active enhancer marks (Acetylation at lysine 27, H3K27ac & Monomethylation at lysine 4, H3K4me1). Notably, SEs have been demonstrated to dictate cell identity and disease and play a key role in carcinogenesis in a broad spectrum of tumors. Recently, it has been proposed that SEs have the potential to serve as valuable prognostic and therapeutic targets in cancer.

During the past decade, various types of cancer pathogenesis have been proved to be closely associated with SEs, such as oncogenes activation, dysregulated signaling pathways, and genetic mutations. Generally, cancer-specific SEs assembly are not presented in the corresponding non-cancerous tissues, such as C-Myc, INSM1 (6) and TAL1 (7). Broadly, these SEs activate the tumorigenic signaling pathways, promoting oncogenic transcriptions, and enriched in key TFs binding motifs. Fortunately, the sensitivity of SEs to perturbation has shown the promising therapeutic vulnerability in various types of cancer, including breast cancer (8), nasopharyngeal carcinoma (9), small cell lung cancer (10), medulloblastoma (11) and esophageal carcinoma (12). The critical oncogenes of ESCC, *e.g. TP63*, *SOX2*, *KLF5* and *ALDH3A1*, have been shown to participate in core regulatory circuitry (CRC) driven by SEs (12). Besides, the pharmacological inhibition of cyclin dependent kinase 7 (CDK7), bromodomain-containing protein 4 (BRD4) and histone deacetylases (HDACs), has been applied in esophageal carcinoma treatment (13).

Therefore, not surprisingly, deregulation of SEs is fundamental mechanism of cancer, which offers an Achilles heel for diagnostic and therapeutic maneuvers (14). This review attempts to discuss SEs’ fundamental characteristics and roles in esophageal carcinoma, which would pave the way for SE-based diagnostic and therapeutic maneuvers.

## Super-enhancers: identification, organization and functions

### Identification of super-enhancers

Enhancers were firstly recognized as the *cis*-regulatory elements from simian virus 40 (SV40), which could prominently promote rabbit β-globulin transcription in HeLa cells (15, 16). In general, enhancers activate cell-type-specific gene expression regardless of their distance, position, and orientation with respective to the cognate promoter (17, 18). The elements of enhancer are bound up with critical TFs through their tissue-specific recognition motifs, thereby functioning as the platform to integrate signaling pathways and further dictate cell lineages (19). Mechanistically, the enrichment of master TFs on enhancers results in recruiting of the subunit of the Mediator complex (Med1), RNA polymerase II (Pol II), and the basal transcription apparatus, which is organized by looping between enhancers and their cognate promoters. Additionally, enhancer regions are mainly overlapped with DNase hypersensitive sites (DHS), and the active state of enhancers are dependent on the following combinations of histone modifications: enrichment with H3K27ac, H3K4me1, and deficiency of Trimethylation at lysine 4 (H3K4me3) (**Table 1**) (20).

**Table 1:** Histone modification markers and the related functional state of the regulatory elements.

Regulatory elements Surrogate Markers	Promoters	Enhancers	Super-enhancers
DHS	+	+	+
BRD4	+	+	+++
MED1	+	+	+++
H3K4me3	+	–	–
H3K4me1	–	+	+
H3K27ac	++	++	+++

DHS, DNase hypersensitive sites; BRD4, Bromodomain-containing protein 4; MED1, subunit of the Mediator complex; H3K4me3, Trimethylation at lysine 4; H3K4me1, Monomethylation at lysine 4; H3K27ac, Acetylation at lysine 27.

SEs were firstly identified as the unique cluster of enhancers in close genomic proximity, which were densely occupied by master TFs Oct4/Pou5f1, Sox2, Nanog (OSN), Klf4, Esrrb, and Med1 in murine embryonic stem cells (mESCs) (21, 22). The Rank Ordering of Super-Enhancers (ROSE) algorithm had been proposed to separate typical-enhancers (TEs) from SEs, whose constituent enhancers were stitched together within 12.5 kb genomic regions enriched by input-normalized level of Med1 signal.

### Structural features of super-enhancers

**Figure 1:**
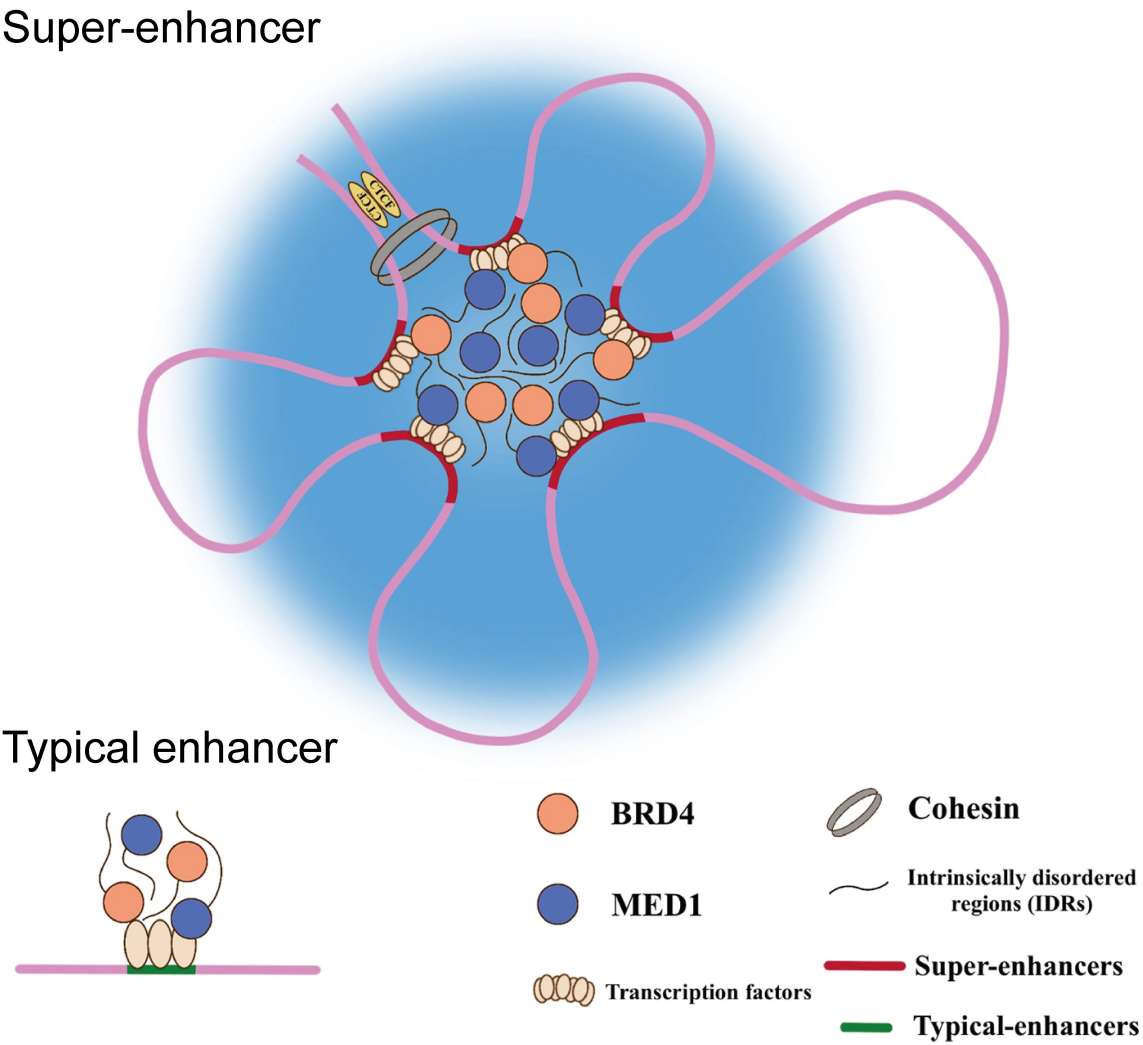
Schematic diagram of the phase-separated condensates at super-enhancers.

Liquid-liquid phase separation (LLPS) is a physicochemical process by which membraneless organelles are generated in eukaryotic cells, which could compartmentalize biochemical reactions within the dense phase (23–25). It has been demonstrated that SEs are phase-separated assemblies accumulated by exceptionally high densities of master TFs, co-activators, and RNA Pol II, dictating the roles in cell identity and disease, including cancers (26). The intrinsically disordered regions (IDRs) of the co-activators (BRD4 & Med1), driven by high-valency and weak-affinity interactions, are responsible for the formation of the phase-separated condensates at SEs, which could bring those *cis*-elements and the cognate promoter in close three-dimensional (3D) proximity and then facilitate SEs activation (**Figure 1**). This physical interaction between enhancer and promoter both involving in TEs and SEs are mediated by cohesin-associated CTCF (CCCTC-binding factor) loops. Additionally, the ubiquitously expressed TF Yin Yang 1 (YY1) has been also identified as the structural regulators contributing to this loop structure (27). The disruption of this structure is likely to result in activation of oncogenes outside the neighborhood by deletion of CTCF binding site, which are consistent with a tendency of liquid phase condensates to undergo fusion.

### Functional properties of super-enhancers

The main features of SEs had been summarized as following Richard A. Young et al. and colleagues: (i) high-density occupancy of master TFs, co-activators and chromatin remodelers (**Table 1**), (ii) large genomic spanning, (iii) ability to exceptionally activate transcription, (iv) sensitivity to perturbation and (v) dictate cell identity and disease (28). Based on these observations, the oncogenic role of SEs usually caused by genetic variations are proposed to drive the transcriptional addiction in cancer.

The transcriptional activities of SEs exhibit an order-of-magnitude higher than TEs and the individual constituent enhancers within SEs, which also showing highly context-dependent manner under rigorous genetic regulation (29). And more interestingly, the cooperativity of SEs constituents is neither additively nor synergistically, suggesting that the “*modus operandi*” of each component under highly precise and complex regulation by other component (30).

## Establishment of oncogenic super-enhancers in esophageal carcinoma

Generally, inappropriate SEs are acquired *de novo* during tumorigenesis compared with the counterpart normal tissues, which driving expression of the critical oncogenes. The malignant transformation and maintenance underpinned by tumorigenic SEs could be seen as one of the core tenets of cancer biology (28, 31, 32). Mechanically, the formation of oncogenic SEs may stem from (i) focal amplification, (ii) genomic rearrangements, (iii) single nucleotide polymorphisms (SNPs), (iv) disruption of topological associating domain (TADs).

### Genetic variations harboring super-enhancers constituents

Somatic copy number alterations (SCNAs) are common mechanism of oncogenesis driven by SEs. For example, focal amplification peak harboring SEs identified by profiling of H3K27ac in esophageal carcinoma (chr13:73880413-74042621, about ~162 kb), which subsequently proven to activate the oncogene *KLF5* expression. And these cancer-specific focal amplification peaks enriched in SEs have also been identified in several types of cancers, including head and neck squamous cell carcinoma, colorectal carcinoma, and liver hepatocellular carcinoma as well (33). Additionally, genomic rearrangements could change the natural genomic context resulting in close proximity between oncogene promoter and their SEs, which ectopically activates gene expression (34). This phenomenon is also described as “enhancer hijacking”, by which oncogenic SEs formed in colorectal carcinoma, medulloblastoma, and acute myeloid leukemia as well (11, 35, 36). Besides, SNPs have been reported to promote tumorigenesis by disrupting the activities of SEs, including acquired oncogenic or abrogating protective allele within master TFs binding sites (37, 38).

These genomic variants provide novel insights into the carcinogenesis of esophageal carcinoma, which deserves further investigation.

### Hijacking of super-enhancers by topological associating domain disruption

TADs have been recognized as the self-interacting genomic region, which demonstrates higher interconnection frequency than the outer regions (39). It has become clear that the main function of TADs is to insulate promoters from distal enhancers or SEs, which conducted by binding of insulator CCCTC-binding factor (CTCF) in cooperation with cohesin complex at their TAD boundaries (40). Although TADs structures are conserved in mammalian, disruption of TADs boundaries caused by genetic or epigenetic could be convenient for abnormal interactions between enhancer/SEs and promoters, which is undoubtedly could be laid the foundation for tumorigenesis.

It has been reported that TADs boundaries disruption could be caused by recurrent mutations of CTCF and cohesin binding sites, which were identified in esophageal carcinoma as well as other types of cancers, including liver hepatocellular carcinoma, colorectal carcinoma, and gastric carcinoma (41, 42). Furthermore, the boundaries disruption is also exemplified by epigenetic dysregulation in glioma by increasing hypermethylation in CTCF site followed by its reduced binding activity (43). Modification of 3D genome by TADs disruption could activate oncogene expression driven by inadvertent SEs-promoter looping, which might be utilized as the novel candidates for targeting the oncogenic SEs in esophageal carcinoma.

## Principles of SEs-driven oncogenic transcription dependencies

### Transcriptional core regulatory circuitries

**Figure 2:**
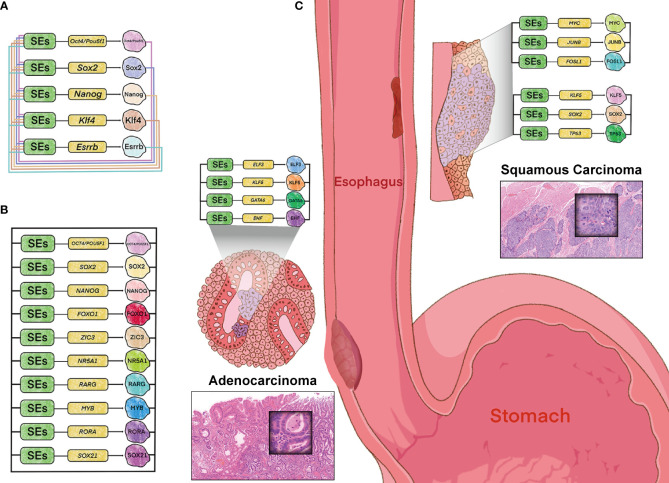
Model of core regulatory circuitry and its implications in esophageal tumorigenesis. **(A)** CRC model in murine embryonic stem cells. **(B)** CRC model in human embryonic stem cells identified by “CRC Mapper” algorithm. **(C)** Histopathology- and cell-type-specific CRC in esophageal carcinoma, including adenocarcinoma (left) and squamous cell carcinoma (right). Hematoxylin-eosin stain (magnification x 100).

The ESC master TFs OSN have previously been demonstrated to bind to their own genes or those of the others in mESCs, which forms an autoregulatory feed-froward loop, *i.e.*, CRC (44, 45). The constituents of this interconnected loop were subsequently extended with the other core TFs, Klf4 and Esrrb, both of which prominent for the maintenance of ESC state (**Figure 2A**) (21). Thus, CRC plays a critical role in the reprogram of somatic cell into induced pluripotent stem cells (iPSCs), and its dysregulation is undoubtedly involved in cancer (12, 46).

Trio-occupancy of OSN has also been well-studied in hESCs (human ESCs), which dominates the pluripotency contributing to human fibroblasts differentiated into the induced pluripotent identity. And this model has been complemented for additional seven key TFs by “CRC Mapper” algorithm, including FOXO1, ZIC3, NR5A1, RARG, MYB, RORA, and SOX21 (**Figure 2B**) (47). The principle of CRC identification by “CRC Mapper” has been characterized as the following three properties: TFs (i) encoded by SEs-assigned genes, (ii) binding to SEs of their own genes, (iii) forming fully interconnected feed-froward loops with other TFs by binding to their SEs.

In accordance with this strategy, cancer-type and -subtype specificity of CRC models have been identified in esophageal cancer and other malignancies. For example, KLF5 has been proven to be collaborated with ELF3, GATA6 and EHF in EAC (46), while cooperated with TP63 and SOX2 in ESCC to form CRC (12), respectively (**Figure 2C**). And SOX2 has also been identified co-regulated with KLF4, EGR1 and NOTCH1 in Glioblastoma (48). These cancer-type and -subtype specific CRCs driven by SEs orchestrate the oncogenic transcriptional addiction, while offers therapeutic vulnerabilities due to perturbation sensitivity of their own SEs. These tissue-specific and cancer-specific CRCs driven by SEs orchestrate the oncogenic transcriptional addiction, which offers therapeutic vulnerabilities consistent with the perturbation sensitivity of their own SEs.

Furthermore, cell-type-specific CRC observed in types of cancers is in line with tumor heterogeneity, one of the hallmarks of malignancy. For example, the other CRC (MYC/JUNB/FOSL1) has been identified in ESCC, different with the KLF5/SOX2/TP63 circuitry (**Figure 2C**). This heterogeneity of CRC coincides with the two major genomic molecular subtypes in ESCC, which are presumably dominated by the two CRCs respectively. And different CRCs have also been identified in other types of cancers, including medulloblastomas and acute myeloid leukemia (49, 50). The heterogeneity of CRC highlights cell-type-specific property of SEs, which sheds light on the novel therapeutic possibilities.

### Concentration of oncogenic signaling pathways on super-enhancers

The constituents within SEs have been demonstrated to be heavily loaded with terminal TFs of the Wnt, Tgfb-1, and Lif signaling pathways in mESCs, showing the preferential affections upon SE-assigned genes compared with genes regulated by TEs (51). Therefore, SEs could serve as a platform to converging multiple signaling pathways, dictating the development and disease state of cells, especially oncogenesis (52). For example, EAC-specific SEs has been proposed to be loaded with tumor-promoting TFs LIF, which contributes its malignant features by activating STAT3 and PI3K/AKT signaling pathways (46). Similarly, the cancer-subtype-specific SEs are densely bound up with its specific master TFs TP63 in ESCC, which participates in the formation of CRC and promotes cancer cell proliferation *via* PI3K/AKT signaling pathways (12, 53, 54). These findings have also been clarified in other types of cancer, e.g., colorectal carcinoma (51), pancreatic carcinoma (55) and osteosarcoma (56). Taken together, these lines of evidence have been proposed that these oncogenic SEs occupied by key TFs pertaining to the critical signaling pathways upon which cancer cell depend, which offers the therapeutic vulnerabilities for esophageal carcinoma.

## The occupancy of CDK7 at super-enhancers in esophageal carcinoma

The cyclin-dependent kinases (CDKs) of mammals comprise two main subfamilies with unique properties associated with cell cycle (CDKs 1-6 & 14-18) and transcriptional regulation (CDKs 7-13 & 19-20) (57). The unique functional repertoire of CDK7, the critical component of CDKs, is based on the regulation of transcription and cell cycle progression. Generally, CDK7 could activate transcriptional initiation and elongation by combining with transcription factor II H (TFIIH), while it could also control cell cycle progression by virtue of forming CDK-activating kinase (CAK) (58, 59).

Studies have demonstrated that the dysregulation of CDK7 was involved in various types of cancers and considered to be positively correlated with the aggressive clinicopathological features of these cancers, including esophageal cancer, hepatocellular carcinoma, gastric cancer, and colorectal cancer (13, 60–62). In ESCC, an immunohistochemical (IHC) analysis demonstrated that elevated expression of CDK7 was observed in over 80% samples which was associated with high tumor grade and poor prognosis. Notably, inhibited proliferation and decreased chemotherapeutic resistance have been observed when CDK7 gene was silenced (63). Besides, the level of CDK7 was higher in ESCC tissues with lymph node metastases compared to control group and positively correlated with tumor metastasis and patients’ overall survival (64).

The overabundance of CDK7 within SEs regions offer the opportunities of blockade therapies in a lineage-specific cancer cell manner. THZ1, a covalent inhibitor of CDK7, was proposed to have a preferential impact on a plethora of oncogenes driven by SEs and could cause the disruption of specific transcriptional programs. For example, Chipumuro et al. found that this inhibitor could suppress the transcription of amplified *Mycn* to suppress the neuroblastoma cells proliferation and the sensitivity to THZ1 was related to preferentially decreased expression of SE-driven oncogenes (65). Subsequent studied showed that esophageal cancer, lung cancer and prostate cancer were sensitive to THZ1 treatment at low nanomolar range (10, 13, 66). Additionally, THZ1 could also cause downregulation of SEs-associated functional long noncoding RNAs acting as competing endogenous (ce-lncRNAs), such as HOTAIR, XIST, SNHG5, and LINC00094 (67), which are associated with expression of cancer hallmark genes. Notably, LINC00094, as a novel oncogenic lncRNA could be activated by master TFs, *e.g.*, TCF3 and KLF5, and positively correlate with poor prognosis. Upon inhibiting these TFs by THZ1, the level of LINC00094 is downregulated, which could cause tumor regression in ESCC. Thus, these studies have demonstrated THZ1 could be utilized as the crosshair of cancer drug discovery (57).

Similar to CDK7, CDK9 is mainly responsible for oncogenic transcription, *e.g.*, as *MCL-1* and *C-Myc*, by binding to elongation complex (p-TEFb) (68) and knocking down CDK9 has shown an excellent antineoplastic activity in hematologic and solid tumors. SNS-032, a selective inhibitor against CDK family as well can inhibit transcription initiation and elongation by targeting both CDK7 and CKD9, with the IC50 values of 62 and 4 nM, respectively (69–71). It could suppress the lung and lymph node metastasis as well as inhibit ESCC proliferation (72). These findings indicate that SNS-032 play an antineoplastic role in ESCC and is expected to enter clinical trials to validate its efficacy, especially in those with metastasis.

## The occupancy of BRD4 at super-enhancers in esophageal carcinoma

The bromo domain and extra-terminal domain (BET) family contain BRD2, BRD3, BRD4, and testis-specific BRDT, which are characterized by acetylation recognition and transcription regulation (73). The well-known member of the BET family, BRD4 was initially recognized as the component of Mediator complex, and subsequently the general regulator for RNA Pol II by virtue of recruitment with transcriptional elongation factor P-TFEb. Consistently, the genomic profiling of BRD4 demonstrated that it is mainly enriched at active enhancers and promoters, which is not surprisingly complicated in critical cellular processes, such as embryogenesis (74) and cancer development (75–82). More importantly, the tumorigenic transcriptional activation of BRD4 are preferentially to regulate SE associated oncogenes, such as *C-Myc* and *BCL2* (83, 84)

Emerging evidence have demonstrated that inhibition of BRD4 occupancy result in SEs disruption and subsequently suppress its related oncogenes expression (73). Up till now, about 20 BRD4 inhibitors have been entered into clinical trials, some of which showed valuable therapeutics for several cancers, including hematological malignancies and non-small cell lung cancer (85). The critical oncogenes within these cancers showed highly sensitivity to JQ1, one of the promising anti-tumor BET inhibitors (22, 86). For instance, JQ1 has been demonstrated to inhibit ESCC proliferation by inhibiting *C-Myc* amplification (86). For another, it could also block recruitment of BRD4 on the promoter of aurora kinases A and B (AURKA/B) to trigger cellular senescence, which provided a novel action manner of BRD4 in esophageal carcinoma (87). In esophageal adenocarcinoma (EAC), high expression of *YAP1* has been reported to be observed and positively associated with poor prognosis. JQ1 is capable of blocking BRD4 binding to the *YAP1* promoter and suppress Hippo/YAP1 signaling (88), which could be synergistically enhanced when traditional chemotherapeutic agents, *e.g.*, docetaxel, are added both *in vitro* and *in vivo* (88). Notably, cell cycle arrest, especially in G1 phase, has been observed in all the studies above. These studies also indicated that JQ1 might be a promising drug in esophageal carcinoma treatment.

Although a variety of small-molecule BET inhibitors have been entered in clinical researches, however, the off-target and side-effects cannot be neglected (89). Proteolysis targeting chimeric (PROTAC) technology have been utilized as an effective degradation tool over the years, which can ubiquitinate the disease-causing proteins by hijacking E3 ligases to achieve the anti-tumor effects (90). Compared to the general small-molecule inhibitors, the hetero-bifunctional molecule based on PROTAC technology demonstrated degradation of BET protein at low nanomolar dose with negligible side-effects (91). The first oral PROTACs (ARV-110, NCT03888612 & ARV-471, NCT04072952) have achieved encouraging benefit for prostate and breast cancer treatment in clinical trials (92). The newly developed GNE987, and PROTAC pan-BET degrader, have been showing good potency against several cancers, including hematological malignancies (93, 94), neuroblastoma (95), and prostate carcinoma (96). Additionally, MZ1, a PROTAC BET degrader, was previously identified unexpectedly degradation of BRD4 over other members of BET family, such as BRD2 and BRD3 (97). However, MZ1 have been proposed to suppress ESCC migration by degrading BRDT, initially recognized as testis-specific protein, rather than BRD2, BRD3 and BRD4. The migratory inhibition of ESCC by MZ1 resulted from down-regulation of *ΔNp63* target genes enriched within SEs (98).

## The enrichment of histone acetylation at super-enhancers in esophageal carcinoma

Histone H3K27ac has been proposed to separate the active enhancers from poised enhancers occupied by H3K4me1 alone (99). The occupancy of H3K27ac facilitates the chromatin accessibility, which is responsible for exceptionally higher enrichment of master TFs, co-activators, and transcriptional machinery on SEs. Therefore, the dysregulated H3K27ac within SEs region could drive the oncogenesis in several types of cancer.

Histone acetylation is a relative steady-state controlled by two families of enzymes: histone acetyltransferases (HATs) and histone deacetylases (HDACs) (100, 101). Numerous studies have demonstrated that the disturbed equilibrium of histones acetylation is closely related with the tumorigenicity of esophageal carcinoma (**Table 2**). Furthermore, the tumor-promoting genes activated by hyperacetylation have also been identified in esophageal carcinoma (**Table 3**). The dysregulated histone acetylation of esophageal carcinoma could be accounted for the oncogenesis driven by SEs, which densely bound up with the active enhancer marker H3K27ac.

**Table 2:** The correlations between dysregulated HDACs (and/or HATs) and the advanced oncogenic features of esophageal carcinoma.

Ref.	Sample info.			Targets	Malignant characteristics
	Pathol.	No.	Cell lines
Langer R et al. (102)	EAC	180	N/A	HDAC2↑	Lymph node metastasis↑+ Pathological Differentiation (poor) (Human Tissues)
Zeng, et al. (103)	ESCC	86	EC/CUHK1, KYSE30/140/150/180	HDAC4↑	Clinical Stage (advanced) + Pathological Differentiation (poor) + OS & PFS (poor) (Human Tissues) Proliferation↑+ Migration↑(*in vitro*)
Xue, et al. (104)	ESCC	167	Eca-109	HAT1↑	Pathological Differentiation (poor) (Human Tissues) Proliferation↑(*in vitro*)
Feng, et al. (105)	ESCC	N/A	Eca-109, EC9706	HDAC7↑	Epithelial-mesenchymal transition↑(*in vitro* & *in vivo*)
Li, et al. (106)	ESCC	N/A	EC9706	HDAC6↑	Proliferation↑+ Migration↑(*in vitro*)

EC, Esophageal carcinoma; EAC, Esophageal adenocarcinoma; ESCC, Esophageal squamous cell carcinoma; HDACs, Histone deacetylases; HATs, Histone acetyltransferases; OS, Overall Survival; PFS, Progress-free Survival.

**Table 3:** The associations between dysregulated histone acetylation modifications and advanced oncogenic features.

Ref.	Sample info.			Targets		Malignant characteristics
	Pathol.	No.	Cell lines	Marker	Genes	
Zhang R et al. (107)	ESCC	90	Eca-109, TE-1	H3K27ac↑	CCAT1↑	Lymph node metastasis↑+ Clinical Stage (advanced) + OS (poor) (Human Tissues) Proliferation↑+ Migration↑(*in vitro* & *in vivo*)
Hu et al. (108)	ESCC	N/A	EC9706, KYSE150	H3K9ac↑	KLF4↑	Proliferation↓(*in vitro*)
Liang et al. (109)	ESCC	115	Eca-109, KYSE150/450	H3K27ac↑	LAMC2↑	Lymph node metastasis↑+ Clinical Stage (advanced) + OS (poor) (Human Tissues) Migration↑+ Invasion↑(*in vitro*)

Based on this knowledge, the aberrant histone acetylation has been addressed as the alternative avenues for cancer treatment, especially those driven by oncogenic SEs. For example, the aberrantly expressed gene *SIRT7*, NAD-dependent deacetylase, was activated by SEs in non-alcoholic fatty liver disease (NAFLD)-associated hepatocellular carcinoma (HCCs). Depletion of *SIRT7* associated SE could suppress the tumorigenicity both *in vitro* and *in vivo* (110). And several types of HDAC inhibitors (HDACis) have been shown as the promising therapeutic strategies against esophageal carcinoma (**Table 4**). Therefore, the recruitment of exceptionally high histone acetylation modification, especially H3K27ac, within the oncogenic SEs could contribute their vulnerabilities to HDACis for patients of esophageal carcinoma.

**Table 4:** The molecular mechanism of histone deacetylase inhibitors against esophageal carcinoma.

Ref.	Sample info.		HDACis				Malignant Features
	Pathol.	Cell lines	Type	Names	Targets	Genes	
Hoshino et al. (111)	ESCC	T.Tn, TE-2	Cyclic Peptides	Romidepsin	HDAC1/2	PRDX1↑+ CDKN1A↑	Proliferation↓+ Apoptosis↑ (*in vitro* & *in vivo*)
Murakami, et al. (112)	ESCC	T.Tn, TE-2	Cyclic Peptides	CHAP31	Pan-HDAC (except HDAC6)	cleaved Caspase-9↑+ Bax/Bcl-2↑	Apoptosis↑ (*in vitro* & *in vivo*)
Tzao, et al. (113)	ESCC	KYSE70/150/510	Hydroxymates	SAHA	Pan-HDAC	CDKs↓+ E-cadherin↑	Proliferation↓ + Migration↓+ Invasion↓ (*in vitro* & *in vivo*)
Shoji, et al. (114)	ESCC	TE-9/10/11/14	Short-chain fatty acids	Valproic acid	HDAC1	RAD51↓	Radiosensitivity↑(*in vitro*)
Shi, et al. (115)	ESCC	TE-1, KYSE510	Hydroxymates	LMK-235	HDAC4/5	TNS3↓	Proliferation↓ (*in vitro* & *in vivo*)
Ma, et al. (116)	ESCC	EC9706, KYSE70	Benzamide	MS-275	HDAC1/3	PI3K/Akt/mTOR↓	Proliferation↓+ Apoptosis↑ (*in vitro* & *in vivo*)
Feingold, et al. (117)	EAC	NCI-SB-Esc1/2/3, OE33, Flo-1	Benzamide	MS-275	HDAC1/3	TXNIP↑	DNA damage↑+ Apoptosis↑(*in vitro*)

HDACis, Histone deacetylase inhibitors.

## Conclusive remark

The concept of SEs and their function in physiology and disease has been established in the last decade. To date, there is no doubt that SEs acquired *de novo* are one of the hallmarks of esophageal carcinoma, and the available studies conclude the characteristics of the oncogenic SEs in esophageal carcinoma: (i) they are acquired based on genetic variants or TADs boundaries disruption, (ii) they drive the transcriptional additions depend on CRCs and convergence of tumorigenic signaling pathways, (iii) they could be targeted by inhibition of BRD4, CDK7, and histone acetylation, which exceptionally enriched within these *cis*-elements. However, there are still several unresolved points regarding SEs in esophageal carcinoma: (i) how the individual *bona fide* constituents selectively respond to targeted inhibition pertaining to the disproportionately enriched factors, (ii) how 3D genome structure of the SEs are influenced by the tumor-promoting signaling pathways, (iii) what are the critical transitions of SEs landscape during the process of precancerous lesions to cancer, (iv) what are the main differences of SEs profiling between squamous cell carcinoma and adenocarcinoma, (v) what are the clinical benefits based on the combinations of traditional treatments and novel therapeutic avenues targeting the subtype-specific or cell-type-specific CRCs. These insights about SEs-driven transcriptional dependencies in esophageal carcinoma, may shed light on the potential clinical application of the cancer-specific SEs.

## Author contributions

BL, HY, and XM formulated the study concept and design; YS wrote the original manuscript draft; MW, DL, provided the technical and material support; SU, critically revised the manuscript. All authors contributed to the article and approved the submitted version.

## Funding

This study was supported by the Zhongyuan talent program (ZYYCYU202012113).

## Acknowledgments

​We apologize to the authors whose study could not be cited for the limited spaces. We would like to thank Jiyu Zhang for his delicate and exquisite schematic graphs. And we also thank Zhixin Li for his comments on this manuscript.

## Conflict of interest

The authors declare that the research was conducted in the absence of any commercial or financial relationships that could be construed as a potential conflict of interest.

## Publisher’s note

All claims expressed in this article are solely those of the authors and do not necessarily represent those of their affiliated organizations, or those of the publisher, the editors and the reviewers. Any product that may be evaluated in this article, or claim that may be made by its manufacturer, is not guaranteed or endorsed by the publisher.
